# Impact of a diabetes disease management program on guideline-adherent care, hospitalization risk and health care costs: a propensity score matching study using real-world data

**DOI:** 10.1007/s10198-022-01486-2

**Published:** 2022-06-18

**Authors:** Marc Höglinger, Brigitte Wirth, Maria Carlander, Cornelia Caviglia, Christian Frei, Birgitta Rhomberg, Adrian Rohrbasser, Maria Trottmann, Klaus Eichler

**Affiliations:** 1 Health Services Research, Winterthur Institute of Health Economics, Zurich University of Applied Sciences, Gertrudstrasse 15, CH-8401 Winterthur, Switzerland; 2 Swica Health Insurance, Winterthur, Switzerland; 3 Medbase Health Care Provider, Winterthur, Switzerland; 4 Institute of Primary Health Care (BIHAM), University of Bern, Bern, Switzerland

**Keywords:** Disease management, Diabetes, Structured treatment program, Primary care, Quality of care, Program evaluation, I10, I18

## Abstract

**Objective:**

To evaluate the impact of a DMP for patients with diabetes mellitus in a Swiss primary care setting.

**Methods:**

In a prospective observational study, we compared diabetes patients in a DMP (intervention group; *N* = 538) with diabetes patients receiving usual care (control group; *N* = 5050) using propensity score matching with entropy balancing. Using a difference-in-difference (DiD) approach, we compared changes in outcomes from baseline (2017) to 1-year (2017/18) and to 2-year follow-up (2017/19). Outcomes included four measures for guideline-adherent diabetes care, hospitalization risk, and health care costs.

**Results:**

We identified a positive impact of the DMP on the share of patients fulfilling all measures for guideline-adherent care [DiD 2017/18: 7.2 percentage-points, *p* < 0.01; 2017/19: 8.4 percentage-points, *p* < 0.001]. The hospitalization risk was lower in the intervention group in both years, but only statistically significant in the 1-year follow-up [DiD 2017/18: – 5.7 percentage-points, *p* < 0.05; 2017/19: – 3.9 percentage points, n.s.]. The increase in health care costs was smaller in the intervention than in the control group [DiD 2017/18: CHF – 852; 2017/19: CHF – 909], but this effect was not statistically significant.

**Conclusion:**

The DMP under evaluation seems to exert a positive impact on the quality of diabetes care, reflected in the increase in the measures for guideline-adherent care and in a reduction of the hospitalization risk in the intervention group. It also might reduce health care costs, but only a longer follow-up will show whether the observed effect persists over time.

## Background

Diabetes mellitus is a tremendous public health issue, and its prevalence is increasing [1]. In Switzerland, the proportion of people with diabetes among over-15-year-olds increased between 2007 and 2017 to 5.4% in men and to 3.5% in women [2]. The treatment of diabetes is complex and requires a careful coordination of measures and of different health professionals providing them. Ill-coordinated care can lead to duplication of services and overuse or, on the other hand, to undertreatment if clinicians do not follow evidence-based guidelines [3, 4]. To overcome poorly coordinated services across involved providers, as well as to strengthen guideline adherence and improve patient outcome, evidence-based disease management programs (DMPs), also called chronic care models or structured treatment programs, have been recommended for the management of patients with chronic conditions such as diabetes [5, 6]. The overarching goal of DMPs is the “optimal” instead of the “maximal” care, being reached by standardization of care and efficient use of resources [5]. The details of a DMP vary between regions and the participating institutions, but it mainly consists of three central parts: (i) evidence-based guidelines (ii) integrated care overcoming barriers between different health professions and institutions to minimize the number of duplicated treatments and (iii) establishment of quality management processes that facilitate the continuous improvement and development of care delivery and guidelines [7].

DMPs have been widely evaluated, but the studies are very heterogeneous [8] and partly used inadequate methodological approaches, such as uncontrolled pre–post-designs [9, 10]. With regard to clinical parameters, a meta-analysis of randomized controlled trials in Europe found only small improvements in the levels of HbA1c, total cholesterol, LDL cholesterol, and blood pressure of diabetes patients in a DMP compared to usual care [11]. As for mortality and costs, a large study that analyzed a nationwide DMP for diabetes in Germany compared intervention to control group, using a propensity score matching strategy. In this 4-year follow-up, the authors found a reduction in overall mortality and in medication and hospital expenditures in the DMP group [12]. Consistently, a systematic review of the effectiveness of DMPs for diabetes patients in Germany concluded that DMPs seem to have a beneficial impact on the mortality and survival time of diabetes patients, but the effects on morbidity, quality of life and monetary outcomes (direct medical costs, cost effectiveness, care expenditures) were inconsistent [13].

For Switzerland, little is known about the impact of structured treatment programs for diabetes mellitus on quality of care and costs in real-world settings. Simulation studies (Markov models) reported that multifactorial interventions (including nephropathy and retinopathy screening, controlling of cardiovascular risk and patient education) may result in yearly savings of 194 million Swiss Francs for the Swiss type 2 diabetes population (285,000 at that time) [14]. A retrospective cohort study using claims data from a large Swiss health insurance company found that the hospitalization risk of diabetes patients was lower if physicians’ guideline adherence was better [15]. Using the same database and a propensity score matching approach, the authors found significantly fewer diabetes-related hospitalizations and lower total healthcare costs (CHF – 778) for patients in integrated care models compared to those in standard models [16]. In addition, an uncontrolled retrospective evaluation of managed diabetes care in a Swiss real-world setting (12 practices from a health provider network) reported improved treatment quality reflected in weight loss, reduction in blood pressure and HbA1c levels [17].

However, evidence for the effect of DMPs in diabetes care in Switzerland is still scarce, particularly with regard to their impact on quality of care [16]. Thus, the aim of this study was to assess the impact of a DMP for diabetes mellitus type 1 and 2 on guideline-adherent care, hospitalization risk (i.e., patient outcome), and health care costs by comparing changes in these outcomes between baseline and years one and two after the introduction of a DMP. Using a difference-in-difference approach with a matched control group (propensity score matching with entropy balancing [18]), our study assesses the impact of the DMP introduction on the intervention group, as far as this is possible using a non-experimental design and real-world data.

## Methods

### Study design and data

We performed a prospective observational study with 2-year follow-up and compared patients with diabetes mellitus enrolled in a DMP with diabetes patients receiving usual care using propensity score kernel matching with entropy balancing [18]. We used a difference-in-difference (DiD) approach [19] and compared changes in outcomes from baseline to 1-year follow-up and from baseline to 2-year follow-up between the DMP (intervention) and the usual care group (control). Analyzing the 1- and 2-year follow-up allows us to assess the robustness of the effect over time and to observe potentially lagged effects of the treatment. The analysis is based on claims data from a large Swiss health insurer (SWICA) with approximately 800,000 insured persons in 2019 (approx. 10% of the Swiss population).

### Study setting

In Switzerland, health insurance is mandatory for every resident. There are several different health insurance providers and health care models to choose from. Various contracted insurance models (mostly with shared saving agreements) exist where physician networks collaborate with insurers. Patients joining such networks get rebates on their insurance premiums. Patients are free to visit all physicians in the standard model, but more than 70% of the population choose a managed-care type contract [20]: in case of illness, these patients are obliged to contact first their GP, a telemedicine center, or a GP of choice within the network, who acts as a gatekeeper to more specialized medical care services. However, structured treatment programs are not implemented on a broader scale in Switzerland. Nevertheless, the ‘Medbase’ health care provider has offered a structured DMP for diabetes in some of its primary care practices since 2017. The present study investigated the effect of this DMP in seven ‘Medbase’ practices in the north-eastern part of Switzerland.

### Participants

In the DMP group (intervention group), we included 538 patients from the SWICA claims database who were identified as having diabetes mellitus type 1 or 2 using pharmaceutical cost groups (PCGs, at baseline) and who were registered in one of the seven ‘Medbase’ practices that introduced the DMP under investigation. “Registered” means that they named a particular practice as their medical ‘home’. Only diabetes patients treated with antidiabetic medication can be identified with PCGs, whereas type 2 diabetes patients without oral drug treatment or insulin cannot be identified and were consequently not included in the study. The control group with usual care (*N* = 5050) consisted of diabetes patients (again identified by PCGs) from the SWICA claims database not participating in a DMP. All participants had to be insured by SWICA over the whole 3-year analysis period. In addition, members of the intervention group had to be registered continuously in one of the practices with DMP.

#### Intervention: disease management program

The DMP under assessment consists of the core elements of a DMP [5]: it is evidence-based, interprofessional, and undergoes continuous evaluation and improvement [21]. Treatment is based on the recommendations of the Swiss society of endocrinology and diabetes (SGED) for the treatment of diabetes mellitus type 2 [22] and a central element is the continuous care by the GP, in collaboration with a medical practice assistant qualified in chronic care. Physiotherapists and nutritionists are involved for all aspects of movement and nutrition, respectively. Continuity of GP care is a central element in primary care that might reduce secondary costs [23]. Regular meetings within professional and practice teams ensure professional exchange and team competence. Current treatment and results of examinations are documented in the electronic medical history and are regularly evaluated together with the patient. Treatment goals and measures are adjusted, if necessary, thus ensuring an individual and tailored patient care. For quality assurance, quality circles are held within the physician network to improve and further develop the treatment concept, based on current clinical performance and prescription data [24].

#### Outcome measures

Guideline-adherent care was assessed using four performance measures that are identifiable in claims data, the “Four simple performance measures (4SPM)” [15]. They include the measurement of HbA1c, lipid profile, and nephropathy status, as well as examination by the ophthalmologist. We slightly adapted the original measures based on the updated Swiss guidelines and on the suggestions of the involved clinicians. Thus, our outcome measures for guideline-adherent care were (a) at least 2 yearly HbA1c measurements or constant glucose monitoring, (b) yearly lipid profile, (c) yearly nephropathy status or an angiotensin-converting enzyme (ACE) inhibitor therapy, and (d) one examination by the ophthalmologist every 2 years. Hospitalization risk, as a proxy for adverse outcomes, was assessed as the share of patients with at least one hospitalization during the considered year. Lastly, the impact of the DMP on health care costs was assessed with the following outcomes: total health care costs (including all types of health care services and pharmaceuticals), outpatient costs (primary and specialized outpatient health care, physio- and ergotherapy, diagnostics and radiology, nutritionists, hospital outpatient services, pharmaceuticals), and inpatient costs (hospitals, excluding rehab and nursing homes). Costs include all billed health care services that are covered by the compulsory basic health insurance policy and are in Swiss Francs (CHF; official 2017 conversion rate to Euros: 0.85; to US$: 1.02; to British £: 0.76).

### Statistical analysis

Difference-in-difference analysis (DiD) [19] was used to determine the effect of the DMP on the outcome parameters. We compare two groups, control and treatment, and two time periods, baseline (2017) and second observation period (2018 or 2019). We independently assess the first-year follow-up (2018 only) as well as the second-year follow-up (2019 only) by comparing them to the baseline year. Analyzing both the 1- and 2-year follow-ups allows us to assess the robustness of the effect over time and to observe potentially lagged effects of the treatment. The estimation equation takes the following form:

$${\widehat{\delta }}_{DD}=\left({y}_{t1}-{y}_{t2}\right)-\left({y}_{c1}-{y}_{c2}\right)$$

where $y$ is any outcome variable, the index t stands for the treatment (DMP) group, *c* for the control group. The index numbers 1 and 2 stand for the baseline period and the second observation period (year 2018 or 2019). ${\widehat{\delta }}_{DD}$ is the DiD estimator and, assuming a correct model, corresponds to the Average Treatment Effect on the Treated (ATT), hence capturing the effect of the DMP. The DiD estimator is positive/negative if, for example, a relative increase/decrease is larger in the treatment group than in the control group.

We estimated the DiD using a propensity score kernel matching approach with entropy balancing to make the treatment and the control group comparable [18]. While propensity score pairwise-matching has been criticized recently as being inefficient and as producing biased effect estimates [25], kernel matching is considered superior to pairwise matching with regard to efficiency, because it makes use of all cases in the control group by weighting them according to their similarity to the treatment cases. Using an optimization function (i.e., a “kernel”), the entropy-balancing approach produces a (weighted) control group with means and variances of the matching variables identical to those of the treatment group. We used an Epanechnikov-kernel with automatic bandwidth selection as suggested by Huber and colleagues [26]. Our matching variables were gender, age-group, region of residence, type of community (urban/rural, size of community), high vs. low deductible, supplementary outpatient insurance and supplementary hospital insurance, and nine PCGs (Pharmaceutical Cost Groups) as indicators for comorbidities from the baseline year (Table 1). We used t-tests and set an alpha level of 5% to test for statistical significance of group differences and of treatment effects. SEs of the DiD estimators were estimated with bootstrapping (500 replications) which allows matching weights to vary between replications, as suggested by Jann [28]. In addition, bootstrapping is advisable due to the highly skewed distribution of the cost differences [27].

**Table 1:** Baseline values 2017: mean values and differences of demographics before (left) and after (right) matching for the control and the treatment group

	Before matching			After matching		
	Control group	Treatment group	Difference	Control group	Treatment group	Difference
Age	67.01	57.26	– 9.74^c^	58.70	57.41	– 1.30
< 20 (0/1)	0.01	0.03	0.02^a^	0.03	0.03	– 0.00
20–39 (0/1)	0.02	0.09	0.06^c^	0.09	0.09	– 0.00
40–59 (0/1)	0.21	0.40	0.19^c^	0.39	0.39	0.00
60–79 (0/1)	0.59	0.42	– 0.17^c^	0.42	0.42	0.00
≥ 80 (0/1)	0.17	0.06	– 0.10^c^	0.06	0.06	0.00
Gender						
Male (0/1)	0.58	0.65	0.06^b^	0.64	0.64	0.00
Region of residence						
Zurich area (0/1)	0.41	0.40	– 0.01	0.41	0.41	0.00
North-Western part of Switzerland (0/1)	0.21	0.13	– 0.08^c^	0.12	0.12	– 0.00
Eastern part of Switzerland (0/1)	0.37	0.47	0.10^c^	0.48	0.48	0.00
Type of community						
Urban, large community (0/1)	0.34	0.32	– 0.02	0.33	0.33	– 0.00
Urban, medium-sized community (0/1)	0.24	0.47	0.22^c^	0.47	0.47	0.00
Urban, small community (0/1)	0.11	0.02	– 0.08^c^	0.02	0.02	0.00
Peri-urban (0/1)	0.20	0.14	– 0.06^c^	0.13	0.13	0.00
Rural (0/1)	0.11	0.05	– 0.06^c^	0.05	0.05	– 0.00
Health insurance						
High deductible (0/1)	0.05	0.12	0.07^c^	0.12	0.12	– 0.00
No supplementary outpatient insurances (0/1)	0.77	0.71	– 0.06^b^	0.71	0.71	0.00
Supplementary hospital insurance: private or semi-private (0/1	0.20	0.11	– 0.09^c^	0.11	0.11	– 0.00
Pharmacy-based cost groups (indicators for comorbidities)						
Diabetes type 1 (0/1)	0.34	0.37	0.03	0.37	0.37	– 0.00
Diabetes type 2 (0/1)	0.19	0.27	0.07^c^	0.27	0.27	– 0.00
Diabetes type 2, hypertension (0/1)	0.46	0.36	– 0.10^c^	0.37	0.37	0.00
Asthma/COPD (0/1)	0.05	0.04	– 0.01	0.04	0.04	0.00
Mental illness (0/1)	0.12	0.07	– 0.05^c^	0.07	0.07	0.00
Chronic pain (0/1)	0.05	0.03	– 0.02^a^	0.03	0.03	0.00
Heart disease (0/1)	0.03	0.01	– 0.02^c^	0.01	0.01	0.00
Glaucoma (0/1)	0.05	0.04	– 0.01	0.04	0.04	0.00
Other PCG groups (0/1)	0.10	0.05	– 0.05^c^	0.05	0.05	– 0.00

538 treatment cases in raw data, 530 treatment cases in the matched sample (8 not matched), 5050 control cases. 0/1 Dummy variables, Values indicate shares of patients

^a,b,c^Statistically significant difference at 5, 1 and 0.1% level, respectively, based on a *t* test

Analyses were performed using the Stata SE 15 software package (StataCorp. 2015. Stata Statistical Software, College Station, Texas, USA) and the KMATCH-ado [28]. To check the impact of outliers in our analysis, we also conducted a 1, 2, and 5% winsorized analysis for the main outcome total cost, setting values of patients with the highest cost changes between the 2 years to the 99th, 98th, or 95th percentile.

## Results

Tables 1 and 2 show background characteristics and outcomes at baseline for the treatment and the control group before and after matching. Before matching, there are significant differences between the groups for most background characteristics. After matching, differences in background characteristics are zero (except for numeric age), and differences in the outcomes are substantially diminished except for a 10 percentage-points higher share of patients with a nephropathy status check (i.e., test for albuminuria) in the treatment relative to the control group.

**Table 2:** Baseline values 2017: mean values and differences of primary outcome variables before (left) and after (right) matching for the control and the treatment group

	Before matching			After matching		
	Control group	Treatment group	Difference	Control group	Treatment group	Difference
Guideline-adherent care						
All four measures fulfilled (0/1)	0.18	0.20	0.01	0.17	0.20	0.03
≥ 2 HbA1c measurements (yearly) (0/1)	0.81	0.81	0.00	0.80	0.81	0.01
Lipid profile (yearly) (0/1)	0.63	0.65	0.02	0.62	0.64	0.02
Nephropathy status (yearly) or ACE (0/1)	0.39	0.47	0.08^c^	0.37	0.47	0.10^c^
Ophthalmologist (every two years) (0/1)	0.67	0.62	– 0.05^a^	0.62	0.62	0.00
Hospitalization risk						
≥ 1 inpatient hospitalization (0/1)	0.24	0.19	– 0.04^a^	0.19	0.19	0.01
Health care costs (CHF)						
Total	11,450	8783	– 2667^c^	9258	8456	– 802
Outpatient	8213	6814	– 1400^c^	7047	6648	– 400
Inpatient (excl. rehab and nursing homes)	1823	1340	– 483^a^	1323	1279	– 44

538 treatment cases in raw data, 530 treatment cases in the matched sample (8 not matched), 5050 control cases. 0/1 Dummy variables, Values indicate shares of patients

*ACE* Angiotensin-converting enzyme, *CHF* Swiss Francs

^a,b,c^Statistically significant difference at 5, 1 and 0.1% level, respectively, based on a *t* test

**Fig. 1:**
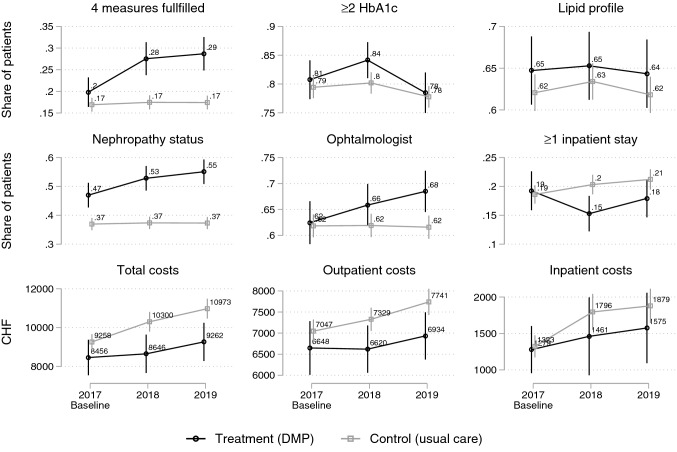
Development of the measures for guideline-adherent care, hospitalization risk, and health care costs by treatment and control group: baseline (2017), 1-year follow-up (2018) and 2-year follow-up (2019). Point estimates with 95% CIs

**Fig. 2:**
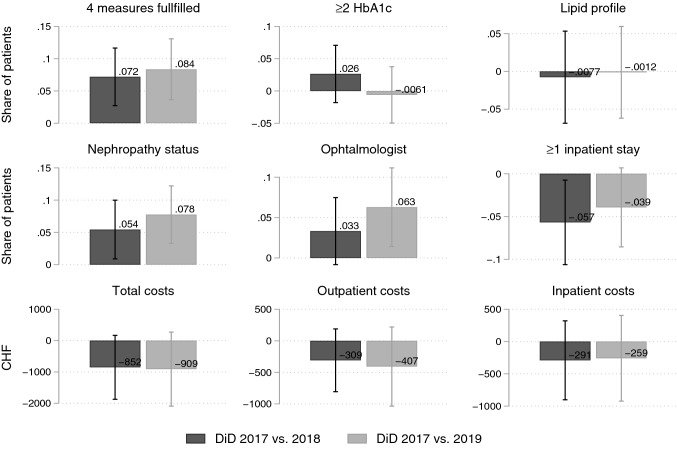
Difference-in-difference estimates of outcomes for baseline (2017) versus 2018 and baseline versus 2019. Point estimates with 95% CIs

Figure 1 shows the outcome trajectories for the baseline year 2017, and for the 2 follow-up years 2018 and 2019. Figure 2 shows the corresponding difference-in-difference (DiD) estimates. The numbers underlying the DiD estimates are presented in Table 3.

**Table 3:** Measures for guideline-adherent care, hospitalization risk, and health care costs: changes and difference-in-difference estimates [95% confidence intervals] from baseline (2017) to 2018 (left) and from baseline to 2019 (right)

	Baseline (2017) versus 2018				Baseline (2017) versus 2019			
	Change in control group	Change in treatment group	DiD estimate	95% confidence interval	Changes in control group	Change in treatment group	DiD estimate	95% confidence interval
Guideline-adherent care								
All four measures fulfilled (0/1)	0.0054	0.077	0.072^b^	[0.027, 0.12]	0.0051	0.089	0.084^c^	[0.036, 0.13]
≥ 2 HbA1c measurements (yearly) or continuous glucose monitoring (0/1)	0.0076	0.034	0.026	[– 0.018, 0.071]	– 0.017	– 0.023	– 0.0061	[– 0.05, 0.038]
Lipid profile (yearly) (0/1)	0.013	0.0057	– 0.0077	[– 0.069, 0.053]	– 0.0026	– 0.0038	– 0.0012	[– 0.062, 0.06]
Nephropathy status (yearly) or ACE (0/1)	0.0041	0.058	0.054^a^	[0.0089, 0.1]	0.0035	0.081	0.078^c^	[0.033, 0.12]
Ophthalmologist (every two years) (0/1)	0.00076	0.034	0.033	[– 0.0083, 0.075]	– 0.0025	0.06	0.063^a^	[0.014, 0.11]
Hospitalization risk								
≥ 1 inpatient hospitalization (0/1)	0.017	– 0.04	– 0.057^a^	[– 0.11, – 0.0074]	0.026	– 0.013	– 0.039	[– 0.085, 0.0069]
Health care costs (CHF)								
Total	1041	190	– 852	[– 1871, 168]	1714	806	– 909	[– 2089, 272]
Outpatient	281	– 28	– 309	[– 807, 189]	694	286	– 407	[– 1034, 219]
Inpatient (excl. rehab and nursing homes)	473	182	– 291	[– 902, 321]	556	297	– 259	[– 923, 404]

538 treatment cases in raw data, 530 treatment cases in the matched sample (8 not matched), 5050 control cases. 0/1 Dummy variables. Values indicate shares of patients

^a,b,c^Statistically significant difference at 5, 1 and 0.1% level, respectively, based on a *t* test

### Guideline-adherent care

In both follow-up years, the share of patients fulfilling all four performance measures increased much more in the treatment than in the control group [DiD 2017/18: + 7.2 percentage-points (95% CI 2.7; 12); DiD 2018/19: + 8.4 percentage-points (95% CI 3.6; 13)]. This finding is due to the treatment groups’ higher increase in the share of patients with yearly examination of nephropathy status or intake of ACE inhibitors [DiD 2017/18: + 5.4 percentage points (95% CI 0.9; 10); DiD 2018/19: + 7.8 percentage-points (95% CI 3.3; 12)] and with ophthalmologic care every 2 years [DiD 2017/18: + 3.3 percentage-points (95% CI – 0.8; 7.5), statistically not significant; DiD 2018/19: + 6.3 percentage-points (95% CI 1.4; 11)]. There were no systematic differences between treatment and control group in the changes in uptake of two or more HbA1c measurements and in lipid profiles (Figs. 1 and 2, Table 3).

### Hospitalization risk

The share of patients with at least one hospitalization per year changed in both follow-ups in favor of the treatment group (Figs. 1 and 2, Table 3): between 2017 and 2018, the control group showed an increase of 1.7 percentage-points in hospitalization risk, the treatment group a decrease of 4 percentage-points, resulting in a DiD of – 5.7 percentage-points in favor of the treatment group (95% CI – 11; – 0.7). Between 2017 and 2019, the increase in the control group was 2.6 percentage-points and the decrease in the treatment group 1.3 percentage-points, resulting in a non-significant DiD of – 3.9 percentage-points in favor of the treatment group (95% CI -8.5; 0.7).

### Health care costs

All cost outcomes showed negative (not statistically significant) DiD estimates from baseline to 1-year follow-up (2018) and 2-year follow-up (2019), a result of smaller cost increases in the treatment compared to the control group (Figs. 1 and 2, Table 3). Total costs, outpatient and inpatient costs increased less in the treatment compared to the control group in both follow-ups, but the differences were not statistically significant. Total costs, for example, increased from 2017 to 2018 in the control group by CHF 1041 (2017/19: 1714) and in the treatment group by CHF 190 (2017/19: 806), resulting in a DiD of CHF – 852 (95% CI – 1871; 168) (2017/19: – 909 (95% CI – 2089; 272)). Results for the winsorized total cost-variable were comparable and even showed statistically significant DiD estimates when winsorizing the 2 or 5% most extreme values, demonstrating that our results are robust and not driven by outlier values.^1^

## Discussion

In this prospective observational study with 2-year follow-up using a difference-in-difference matching approach, we evaluated the impact of a DMP introduction for diabetes patients in Switzerland on guideline-adherent care, hospitalization risk and health care costs compared to usual care. Adherence to treatment guidelines improved in the treatment group, particularly for the examination of nephropathy status (or intake of ACE inhibitors) and for ensuring regular ophthalmologic examinations. The hospitalization risk, too, changed in favor of the treatment group, indicating that also patients’ health status benefited from the DMP. Health care costs increased substantially less in the treatment compared to the control group. Although this difference was not statistically significant, it accounts for about 10% of the total annual health care costs of CHF 8456 in the intervention group and CHF 9258 in the control group (after matching).

In line with this study’s finding of increased guideline adherence and a decrease in hospitalization risk after introducing the DMP, Huber and colleagues [16] found a reduced probability of future hospitalizations for patients in an integrated care model compared to standard care (OR of 0.87; 95% CI 0.79; 0.95). The same authors also reported a clear link between hospitalization risk and physicians’ guideline adherence as measured by the 4SPM [15]. Annual health care costs in our sample of diabetes patients are in a similar range to those found in a study by Huber and colleagues [16], who used claims data of another large Swiss health care insurer for the year 2013 and reported mean annual costs of CHF 9466 for diabetes patients in an integrated care model vs. CHF 10,530 for patients in a standard care model. In addition, the (cost-saving) effect of CHF – 778 for the integrated care model for diabetes patients that they report is similar in range to the effect of the DMP in our study. This is notable, as the two studies used data from different Swiss health insurers, from different years, and they used a somewhat different methodology: pairwise propensity score matching and regression adjustment vs. propensity score kernel matching with entropy balancing combined with difference-in-difference in our study.

### Strengths and limitations

The major strength of our study is the analysis of both the quality of care (i.e., guideline adherence and hospitalization risk) and the resulting health care costs. The simultaneous assessment of patient benefit and costs is essential to gain a better understanding of the real value of health care for patients [29]. Furthermore, we analyzed data of a large Swiss health insurer, which adds evidence about the real-world impact of a structured diabetes care approach in primary care in a social health insurance system. Our study has, however, several limitations. It is an observational study and causal inference can, strictly speaking, not be drawn. We addressed the problem of confounding using a DiD approach that removes baseline differences between the treatment and control groups. Using propensity score matching based on entropy balancing, we made the groups comparable with regard to age, gender, comorbidities and place of living. Still, a selection bias may remain because unobserved differences likely influenced the probability of patients being part of the treatment or the control group. A further limitation is that we used pharmacy-based indicators (PCGs) to identify diabetes patients in the claims data and that we had no clinical data about the diagnosis, such as the diabetes type or severity. While PCG-based indicators are widely used and have been shown to be quite valid morbidity measures [30], diabetes patients without antidiabetic therapy are consequently not included in this study. We also do not know whether the diabetes patients enrolled in a practice offering the DMP under evaluation did in fact take full part in the DMP, i.e., made use of all the offered services and consultations. However, as this reflects the “real-world”-situation, our analysis represents the true impact of the DMP under analysis even better.

## Conclusion

The DMP under evaluation seems to lead to a better quality of diabetes care at lower health care costs. This has implications for clinicians and managers of health care organizations alike. However, the cost differences are not statistically significant, and the follow-up is short. If the results can be confirmed in a longer follow-up, such structured treatment programs are a good example of value-based health care, as they provide better quality of care at similar or—possibly—even at lower costs.

## Data Availability

The claims data were provided by the SWICA Health Insurance Company, Winterthur, Switzerland. It is not publicly available due to privacy concerns and legal restrictions.

## Footnotes

1 The DiD for the 2% winsorized total costs 2017 vs. 2018 was CHF – 833 [96% CI – 1582; – 84], for the 5% winsorized total costs CHF -849 [– 1410; – 288]). The corresponding values for 2017 vs. 2019 were CHF – 803 [– 1664; 59] and CHF – 712 [– 1373; – 52], respectively. The winsorized estimates are more likely to reach a specified statistical significance level because winsorizing reduces the variance and, consequently, the standard errors of the estimates.

## Code availability

Analysis code available on request.
